# Acupuncture therapy for preventing the nausea and vomiting following high emetic risk chemotherapy: A protocol for systematic review and Bayesian Network meta-analysis

**DOI:** 10.1097/MD.0000000000022150

**Published:** 2020-09-18

**Authors:** Yi-ran Deng, Cheng-wei Fu, Tong Wu, Wan-ping Huang, Hong Nie, Yang Jiao

**Affiliations:** a Hubei University of Traditional Chinese Medicine, Wuhan; b The Second Clinical College of Guangzhou University of Chinese Medicine, Guangzhou; c Wuhan The 9th Hospital; d Hubei Provincial Hospital of Traditional Chinese Medicine; e Hubei Province Academy of Traditional Chinese Medicine, Wuhan, China.

**Keywords:** acupuncture-related therapy, chemotherapy-induced nausea and vomiting, network meta-analysis

## Abstract

**Background:**

Nausea and vomiting are the most common complications after chemotherapy, which cannot be completely controlled even with commonly prescribed antiemetic drugs, particularly in patients receiving highly emetogenic chemotherapy Acupuncture therapy is an effective replacement method for chemotherapy-induced nausea and vomiting (CINV), which effectiveness and safety have been observed by many clinicians. However, different acupuncture treatments have various effectiveness. Based on enough clinical researches, the study aims to uses Bayesian network meta-analysis (NMA) to evaluate the effectiveness of different acupuncture therapies used for preventing CINV.

**Methods:**

Authors will search PubMed/Medline, Cochrane library, Web of Science, Ebsco, Ovid/Embase, China National Knowledge Infrastructure, Wanfang Database, VIP Database, and China Biology Medicine from setup time to July 2020. All randomized control trails meet the standard will be included. Quality evaluation of included studies will be implemented with Cochrane risk-of-bias tool. STATA 14.0 will be used to perform pairwise meta-analysis. Addis 1.16.8 (University Medical Center Groningen (UMCG), Groningen, NL) and OpenBUGS 3.2.3 (Medical Research Council (MRC), London, UK) will be used to conduct NMA.

**Results:**

The results of this review will generate a comprehensive review of current evidence and be published on a peer-reviewed journal.

**Conclusion:**

The result of this systematic review and Bayesian NMA may offer better options for patients in relieving CINV.

Systematic review registration number: INPLASY202070070

## Abstract 2

Supplemental Digital Content is available in the text

## 1 Introduction

High emetic risk is defined as a risk of vomiting within the first 24 hour after start of chemotherapy of >90% in patients who do not receive prophylactic antiemetics,^[1]^ which includes cisplatin, mechlorethamine, streptozocin, cyclophosphamide > 1500 mg/m2, carmustine, dacarbazine, and the combination of an anthracycline and cyclophosphamide (AC).^[2]^ Despite important advances in new and effective preventative antiemetics, chemotherapy-induced nausea and vomiting (CINV) remains among the most unpleasant and feared side effects of cancer chemotherapy.^[3]^ As a prognostic factor for overall survival, it impacts not only the patient's quality of life, but also treatment outcomes.^[4]^

The main pharmacologic classes of drugs used in preventing and treating CINV are 5-HT3 receptor antagonists, Neurokinin-1 receptor antagonists, and corticosteroid.^[5]^ However, antiemetic drugs reduce vomiting and sickness accompanied by many side effects such as constipation, headaches, hiccups, and so on.^[6]^

Thus, Researchers and patients are looking for an additional methods of controlling CINV, such as nondrug therapies. Historically, acupuncture has played an important role in protecting the health of Chinese people for more than 2000 years and is increasingly gaining popularity in some western countries,^[7,8]^ which effectiveness and safety have been admitted by National Institutes of Health (NIH) Consensus Statement.^[7,9]^ The Oncology Nursing Society also considers acupoint stimulation as a promising intervention for the management of CINV.^[10]^ There are kinds of acupuncture therapies to treat CINV, such as moxibustion, acupressure, electric acupuncture. These acupuncture therapies have been shown to be effective in randomized controlled trials (RCTs)^[11–13]^ However, there are no related report about the comparison among different acupuncture therapies. In this study, we aim to evaluate the effectiveness between different acupuncture therapies using network meta-analysis (NMA) based on a Bayesian model.

## 2 Methods

The protocol has been funded and registered on INPLASY (https://inplasy.com/) with the registered ID INPLASY202070070. We used the Preferred Reporting Items for Systematic review and Meta-Analysis Protocols statement.^[14,15]^ Because this is a systematic literature research, ethical approval can be skipped.

### 2.1 Eligibility criteria

#### 2.1.1 Type of study

Only peer-reviewed RCTs will be eligible for inclusion. And language will be restricted to English and Chinese. Conference papers, review, case report, protocol, animal study, supplementary issue, comments will be excluded.

#### 2.1.2 Participants

Adult patients who are diagnosed with neoplasm and receiving highly emetogenic chemotherapy regimens will be included. Patients receiving chemotherapy for blood or rheumatic diseases will be excluded.

#### 2.1.3 Interventions

**Figure 1:**
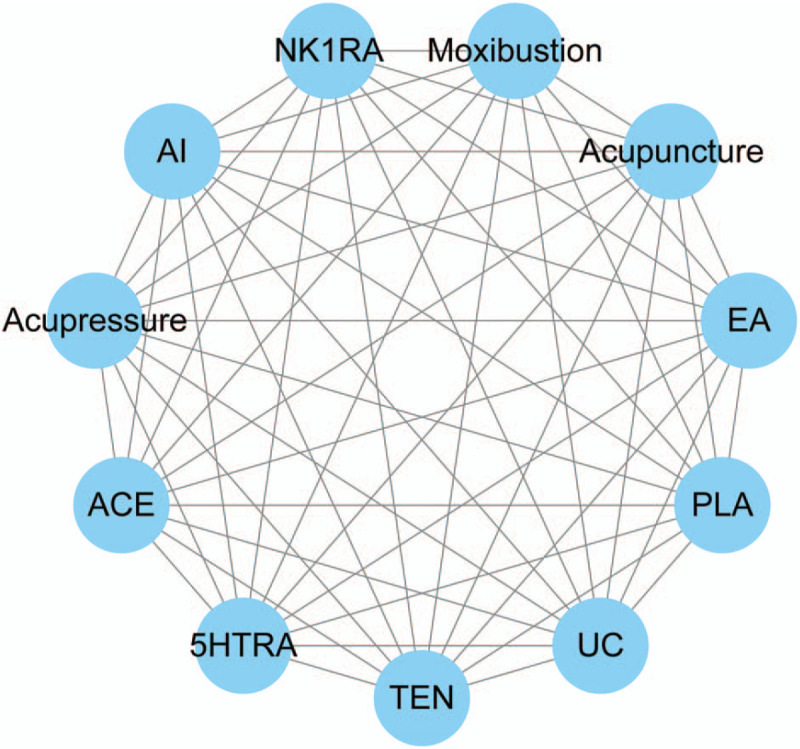
Network plot of possible direct comparisons. (TEN, transcutaneous electrical stimulation; EA = electro-acupuncture, AI = acupoint injection, ACE = acupoint catgut embedding, 5HTRA = 5-hydroxytryptamine receptor antagonist, D2RA dopamine D2 receptor antagonist, PLA = placebo, UC = usual care).

Studies in which acupuncture therapies are performed as interventions will be included, for instance, acupuncture, electro-acupuncture, moxibustion, acupressure, catgut embedding, etc. Acupuncture therapy combined with commonly prescribed antiemetics or usual care will also be recorded while studies whose experimental groups contain other therapies like herbs or massage will be excluded. Figure 1 gives example to illustrate a potential network plot.

#### 2.1.4 Control Group

Control group consisted of usual care (means no treatment), sham acupuncture therapy, medication such (e.g., 5-hydroxytryptamine receptor antagonists or proton pump inhibitors or corticosteroids) will be included. But other complementary or alternative therapies will be excluded (e.g., psychological guidance or herbs)

#### 2.1.5 Outcomes

##### 2.1.5.1 Primary Outcomes

Main outcome will be the incidence of nausea and vomiting, in which 0 to 24 hours after chemotherapy is defined as acute vomiting, while greater than 24 hour is defined as delayed vomiting.

##### 2.1.5.2 Secondary Outcomes

The secondary outcomes will be the grade of nausea and vomiting, Index of Nausea and Vomiting and Retching International Scale or any other clinical assessments.

### 2.2 Search strategies

Authors will search PubMed/Medline, Cochrane library, Web of Science, Ebsco, Ovid/Embase, China National Knowledge Infrastructure, Wanfang Database, VIP Database, and China Biology Medicine disc from setup time to July 2020.

The search strategies will contain both CINV and acupuncture therapy. Items including chemoembolization, chemotherapy, Chemical therapy nausea, vomiting, and similar terms and will be used to identify CINV. “Acupuncture”, “moxibustion”, “acupressure” and similar terms will be used to identify acupuncture therapy. Search strategy will be adjusted according to various databases. Supplemental Digital Content (Appendix 1, http://links.lww.com/MD/E841) shows the detailed search strategy of PubMed/Medline.

### 2.3 Study selection

**Figure 2:**
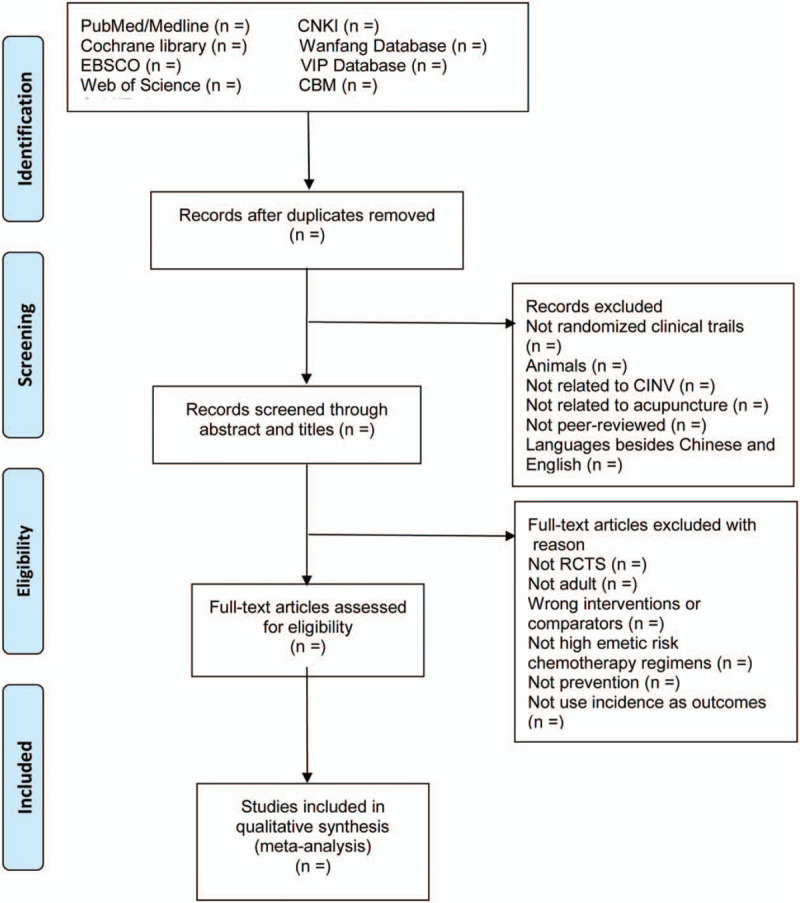
PRISMA flow diagram of the study selection process.

In order to ensure high inter-rater reliability, a predefined inclusion and exclusion criteria will be used. Two reviewers (Tong Wu, Yi-ran Deng) will scan all studies independently according to Supplemental Digital Content (Appendix 2, http://links.lww.com/MD/E842) and a third reviewer (Yang Jiao) will request adjudications if necessary. Only the most informative and complete study of any duplicate publications will be selected. The process of screening will be shown by PRISMA flow diagram as Figure 2.

### 2.4 Data extraction

After identifying the target RCTs, 1 reviewer (Cheng-wei Fu) will extract the following data into a database created by Excel 2019 and checked by the second reviewer (Wan-ping Huang):

(1) literature information: title, first author, publication year, first author's country, ethical approval, and registration of clinical trial registry;
(2) patient information: sample size, sex, diseases, chemotherapy regimens, antiemetic drugs, type of cancer, types of intervention, types of symptoms, time of acupuncture intervention, and acupoints;
(3) outcomes information: the incidences of nausea and vomiting. The third reviewer (Yang Jiao) is the referee in case of doubts or disagreements. In addition, Get Data Graph Digitizer will be employed to extract the number if data are presented as figures.

### 2.5 Risk of bias assessment.

Cochrane risk-of-bias tool (ROB 2.0 (Centre for Evidence-Based Medicine Odense (CEBMO), Odense, DK)) will be used to evaluate the quality.^[16]^ There are 5 sources of bias including:

(1) bias arising from the randomization process,
(2) bias due to deviations from intended interventions,
(3) bias due to missing outcome data,
(4) bias in measurement of the outcome,
(5) bias in selection of the reported result. Finally, an overall risk of bias will be given based on above bias. Two reviewers (Yi-ran Deng and Hong Nie) will use ROB 2.0 to assess all matched studies and the third reviewer (Tong Wu) will request adjudications if necessary.

### 2.6 Statistical analysis

#### 2.6.1 Pairwise meta-analysis

Only 3 or more studies comparing same interventions directly will be conducted in pairwise meta-analysis. Authors will use Stata 14.0 to solve pairwise meta-analysis, odds ratio (OR) and 95% confidence interval (CI) will be adopted. Heterogeneity is quantified with the I^2^ statistic. When I^2^ > 50%, a random effect model will be adopted; if not, a fixed effect model. And before selecting model, sensitivity analysis will be accomplished if sufficient studies are available. When pairwise comparison studies ≥10, a Begg testing will be performed to explore the publication.

#### 2.6.2 Network meta-analysis

NMA is the development of traditional meta-analysis. In this study, Addis1.16.8, OpenBugs3.2.3 and Stata14.0 will be performed to present a NMA. OR and 95%CI will be adopted in the light of incidence, the outcome indicator, as dichotomous data. As to inconsistency test for results, we will monitor the loop formed by studies with both direct evidence and indirect evidence, to figure out whether the IF value approximate 0. With a 95%CI, it indicates a slim possibility of inconsistency if 0 is included. Besides, the surface under the cumulative ranking curve values will carry out a possible range of interventions results, from 0% to 100%. The closer the value is to 100%, the worse the intervention effect will be.

#### 2.6.3 Quality of evidence

Quality of evidence will be evaluated by the Grades of Recommendations Assessment Development and Evaluation (GRADE) guidelines. There are 3 factors (residual confounding, dose-response gradient and large magnitude of effect) to promote the quality and 5 factors (study limitations, inconsistency, indirectness, publication bias, and imprecision) to lower it and the quality will be graded in very low, low, moderate and high. GRADE profiler 3.6 will be used to conduct the assessment.

### 2.7 Subgroup analysis

If 1 of the outcome parameters demonstrates statistically significant differences between intervention groups, we will plan to use subgroup analysis. Planned subgroup analysis will be performed in types of symptoms, types of chemotherapy drugs and so on.

## 3 Discussion

As a routine treatment for cancer patients, the incidence of CINV is prone to metabolic disorders, malnutrition, and weight loss, which will lead to an obvious negative effect on patients’ emotion, social, and physical function. CINV is related to a variety of factors, such as female, a history of nausea and vomiting, anxiety, fatigue, motion sickness, poor quality of life, and low alcohol intake.^[17]^ Even when prophylactic antiemetics are recommended in the guidelines, about 28% of patients with high or moderate emetogenic chemotherapy fail to achieve complete remission.^[18]^ Therefore, it is necessary to explore complementary therapy to prevent CINV, especially for the patients suffering from nausea and vomiting induced by the highly emetogenic chemotherapy.

Some low to moderate evidences have shown the effectiveness and safety of acupuncture therapy for the prevention of CINV,^[19,20]^ but it lacks studies which compare different acupuncture therapies, so that clinicians cannot judge the therapeutic value of different forms of regimens, which is not beneficial for them to choose the best acupuncture treatment. Our research aims to provide a clinically useful ranking of acupuncture interventions for CINV prophylaxis, as well as to provide credible evidence for initiative research directions.

Despite these efforts, limitations in this systematic review will still exist. For example, we will only search English and Chinese databases, which will lead to some biases. Additionally, as there are different types of chemotherapy regimens, different types of nausea and vomiting such as acute, delayed, and postoperative nausea and vomiting, thus heterogeneity may be produced inevitably. Various levels of nausea and vomiting are used in post-treatment management, such as the World Health Organization (WHO), European Clinical Academic Conference Standards and NCI-CTCAE standards, may also contribute to some troubles in data analyzation. Therefore, subgroup analyses will be carried out around these contents to reduce the inconsistency. The results of this protocol will be published in related journal including neoplasms, chemotherapy regimens, complementary and alternative therapy, and a quick update will be made if supplements are required.

## Author contribution

**Data curation:** Cheng-wei Fu, Yi-ran Deng, Yang Jiao.

**Formal analysis:** Tong Wu, Wan-ping Huang, Hong Nie.

**Funding acquisition:** Yang Jiao.

**Investigation:** Yi-ran Deng, Wan-ping Huang.

**Methodology:** Cheng-wei Fu, Yi-ran Deng.

**Original draft:** Cheng-wei Fu, Yi-ran Deng.

**Project administration:** Yang Jiao.

**Resources:** Cheng-wei Fu, Yi-ran Deng.

**Review & editing:** Cheng-wei Fu, Yi-ran Deng.

**Software:** Cheng-wei Fu, Hong Nie.

**Supervision:** Yang Jiao.

## Abbreviations

Abbreviations: CI = confidence interval, CINV= chemotherapy-induced nausea and vomiting, NMA = network meta-analysis, OR = odds ratio, RCTs = randomized control trails.
